# Help-seeking for mental health: an exploratory cross-national study of Italian and South Korean university students

**DOI:** 10.3389/fpsyg.2026.1839818

**Published:** 2026-08-21

**Authors:** Anna Joy Russ, Serena Petrocchi, Yungwook Kim, Peter J. Schulz

**Affiliations:** 1 Faculty of Communication, Culture and Society, Università della Svizzera Italiana, Lugano, Switzerland; 2 Faculty of Biomedical Sciences, Institute of Family Medicine, Università della Svizzera Italiana, Lugano, Switzerland; 3 Division of Communication and Media, Ewha Womans University, Seoul, Republic of Korea; 4 Wee Kim Wee School of Communication and Information and LKC School of Medicine, Nanyang Technological University (NTU), Singapore, Singapore

**Keywords:** cross-national, help-seeking, mental health, self-construal, self-stigma, social support

## Abstract

**Introduction:**

Mental health help-seeking intentions are associated with individual, social, and contextual factors, yet cross-national research comparing Western and East Asian populations remains limited, and few studies have evaluated whether cultural variables contribute explanatory value beyond established predictors.

**Methods:**

A quantitative cross-sectional survey was administered to 304 university students (154 Italian, 150 South Korean) aged 19–35. A series of block-wise hierarchical regressions examined associations between demographic characteristics, social support, self-stigma, prior service use, and cultural variables with help-seeking intentions in two independently recruited national samples.

**Results:**

Support from significant others toward mental health services was associated with intentions in early model steps but lost significance when cultural variables were added. Prior experience with mental health services was positively associated with future intentions. Among the variables entered in the final model, nationality had the largest standardized regression coefficient (β = 0.251), with South Koreans reporting higher observed scores than Italians. Resistance to change-beliefs was positively associated with intentions, though this finding should be treated with caution given measurement non-equivalence. Independent and interdependent self-construal contributed no significant explanatory variance.

**Discussion:**

These exploratory findings suggest that nationality and individual-level cultural proxies may capture distinct dimensions of cultural associations and point to promising directions for future research on culturally adapted mental health interventions.

## 1 Introduction

Seeking professional support for mental health concerns is a complex behavior shaped by an interplay of demographic, psychological, and cultural factors. Despite growing awareness around psychological wellbeing, many individuals remain reluctant to seek help, especially among young people (Doan et al., 2020; Essau, 2005).

While there is no universally accepted definition of help-seeking behavior, increased efforts to fill this gap are quickly expanding (Heerde and Hemphill, 2018). Help-seeking behavior pertains to an individual's actions or attitudes when seeking assistance or support to address a challenge or achieve a goal (Shimada et al., 2021). Help-seeking has been examined within the mental health context, including both turning to family and friends and seeking support from mental health professionals. According to Rickwood et al. (2005), help-seeking for mental distress involves actively reaching out to others for support. A key aspect of this process is the involvement of external sources of support, whether they are close personal contacts or more distant figures. Data indicates a widespread reluctance to seek formal help, with only a minority of individuals accessing professional services even when experiencing significant psychological distress (Essau, 2005). Addressing this issue requires a deeper understanding of the determinants that shape help-seeking intentions.

One of the most established theoretical frameworks for understanding healthcare service utilization is the Andersen Health Care Utilization Model (Andersen, 1995; Andersen and Newman, 1973). This model offers a framework to explain a person's choice to utilize healthcare services. The model states that demographic characteristics, such as people's propensities to utilize services, resources that facilitate or obstruct use, and their need for treatment, can predict or explain how often people use these services (Isaak et al., 2020). The model posits that health service care use is determined by three main components: predisposing characteristics (such as age, gender, and beliefs), enabling factors (such as social support and resources), and need-related variables (such as perceived or evaluated need for care) (Andersen and Newman, 1973). These crucial components have an impact on a person's propensity to seek medical care, including mental health services (Andersen and Newman, 1973; Alkhawaldeh et al., 2023). Enabling resources, both informal and formal, can help or hinder people from using health services. They are founded on the idea that, even if someone is predisposed to do so, they still need to have the means and knowledge necessary to access and use those services. Moving on to the needs, the model recognizes two types of needs for treatment: evaluated need and perceived need. Perceived need is a person's perception of their health and the severity of their symptoms, whereas evaluated need is a professional medical measurement of their health condition and their need for health care (Andersen and Newman, 1973). Help-seeking behavior and use of medical services can be affected by both types (Alkhawaldeh et al., 2023; Isaak et al., 2020).

Drawing from this framework, the current study considers gender, marital status, and age as key predisposing characteristics, while social support and self-stigma are conceptualized as enabling factors that may be associated with individuals' intentions to seek psychological help. Prior experience with mental health services is included as a history-of-care variable. In the original Andersen model, need is ideally operationalized using current perceived or evaluated health status. The present study did not include direct measures of current psychological distress. Prior mental health service use was therefore used as an imperfect proxy for history-of-care, rather than as a direct measure of the model's need component. Beyond these, the present study proposes a cultural extension to the model, introducing nationality, self-construal, and resistance to change-beliefs as a fourth explanatory block.

Among the predisposing characteristics, gender consistently emerges as having a significant association with help-seeking behavior. Numerous studies have shown that women are more likely than men to express positive attitudes toward mental health services and to seek professional support (Abdelmonaem et al., 2024; Möller-Leimkühler, 2002; Qiu et al., 2024). This discrepancy has been linked to a variety of factors, including gender socialization, stigma sensitivity, and cultural expectations surrounding emotional expression. Conversely, men may underutilize mental health care due to concerns about appearing weak, self-reliance norms, or internalized stigma, all of which can create additional barriers (McKenzie et al., 2022). Beyond help-seeking intentions, gender may also shape other psychological and cultural variables associated with mental health behavior. For instance, prior research has suggested that women report higher levels of perceived social support and lower levels of resistance to change, while men may experience greater self-stigma (Corrigan and Watson, 2002; Vogel et al., 2011). Moreover, gender may change how individuals relate to cultural dimensions such as independence and interdependence, with women often endorsing more interdependent values. For this reason, the present study also explores gender differences across the individual and cultural variables of interest, and whether these patterns contribute to understanding broader help-seeking behaviors.

In terms of enabling factors, social support and self-stigma both have been found to play important roles in the definition of help-seeking behavior. Firstly, social support can lower psychological barriers to help-seeking and promote recognition of mental health needs. It has been associated with more positive attitudes toward professional services and a greater likelihood of initiating treatment (Vogel and Wei, 2005; Pescosolido and Boyer, 2010). This study assesses both general support and support specifically related to psychological counseling. In addition to general social support, the current study contributes to the literature by adding a further facet of support: the one that significant people give toward mental health services. The opinions and expectations of significant others, whether supportive or stigmatizing, can powerfully shape help-seeking behavior (Roh et al., 2015). Social interactions and interpersonal relationships have a profound impact on individuals' help-seeking decisions, as they can either facilitate or hinder the initiation of therapy. Supportive and encouraging attitudes from significant others, who may recognize the importance of mental health and endorse professional help, have been found to positively influence an individual's intention to seek therapy (Seyfi et al., 2013). Self-stigma, on the other hand, refers to the internalization of negative societal beliefs about mental illness, leading individuals to judge themselves harshly and to avoid seeking help due to fear of shame or rejection (Corrigan and Rao, 2012). Self-stigma is associated with delayed treatment, reduced adherence to care, and increased psychological burden. It can act as a significant barrier even when external resources are available and supportive (Rüsch et al., 2005).

Despite the Andersen Health Care Utilization Model extensive application in physical health contexts, its use in predicting mental health help-seeking intentions—particularly across culturally diverse populations—remains less explored (Babitsch et al., 2012; Radhamony et al., 2024; Krzyż et al., 2023). To address this gap, the present research integrates the Andersen model with cultural psychological constructs to examine how individual, social, and cultural dimensions are jointly associated with mental health help-seeking intentions. While the Andersen model accounts for many important predictors, it does not explicitly incorporate cultural variables. Yet cultural context plays a central role in shaping how individuals understand mental health, interpret distress, and decide whether and how to seek support (Hwang et al., 2008). Most cross-national studies have focused on comparisons between Western and Eastern cultures, revealing differences in emotional expression, wellbeing, and identity formation (Lim, 2016; Joshanloo, 2014). However, few studies have explored how these cultural differences manifest in mental health help-seeking behavior. Cultural factors significantly shape individuals' attitudes, beliefs, and behaviors not only toward health in general, but also in relation to mental health [Levesque and Li, 2014; Office of the Surgeon General (US), Center for Mental Health Services (US), and National Institute of Mental Health (US), 2001]. Nevertheless, the predominant focus of studies has been on either Western or Eastern cultures separately (see Foster et al., 2021 and Yeh, 2002).

Studies conducted across diverse cultural contexts highlight a range of interpretations regarding the influence of culture on mental health help-seeking, often yielding mixed and sometimes contradictory results. Cultural values may shape help-seeking behavior in complex and sometimes contradictory ways. In collectivistic cultures, where group interdependence is prioritized (Gambrel and Cianci, 2003), seeking professional help for mental health issues may be seen as a sign of weakness or as a burden on the group, leading individuals to rely on informal support sources instead (Leong and Lau, 2001). Yet, collectivistic values can also facilitate help-seeking when it is framed as a means of maintaining one's contribution to family or community wellbeing (Taylor et al., 2004; Triandis, 2018; Yang and Kleinman, 2008). Conversely, individualistic cultures may see professional help as an expression of personal autonomy (Santos et al., 2017; D'Amico and Scrima, 2016); while simultaneously presenting challenges through smaller social support networks and reduced interpersonal embeddedness (Kito et al., 2017; Scott et al., 2004).

To capture the cultural dimension of help-seeking behavior, the present study includes three distinct variables, each representing a different way of conceptualizing culture. Nationality is included in the primary analyses as a sample-membership indicator that distinguished the two independently recruited groups. Self-construal and resistance to change-beliefs are included as exploratory cultural proxies. Self-construal has been applied to cultural psychology in different studies related to mental help-seeking (Yeh, 2002; Omori, 2007). The core idea is that individualistic cultures are characterized by a strong independent self-construal, where individuals value their actions and feelings, while interdependent self-construal is prevalent in collectivistic cultures, where social ties are much more meaningful (Markus and Kitayama, 1991). Resistance to change-beliefs is included as a less conventional cultural variable. Though rarely studied in relation to mental health, it reflects an individual's broader worldview, including openness to novelty, adherence to tradition, and attitudes toward change—factors that have been connected to conservative ideological systems and that could meaningfully shape one's willingness to seek professional psychological support (Jost et al., 2004; White et al., 2020). Cultural norms that emphasize stability, conformity, and traditional gender roles may discourage individuals from seeking external support (Okafor et al., 2022), although there is still no definitive evidence. These three variables capture different aspects of the sample: nationality reflects sample-membership, while self-construal and resistance to change-beliefs represent individual-level proxies (Schwartz, 2014).

In terms of population, this study compares two distinct ones: Italians and South Koreans. Italian culture is characterized by strong family ties, emotional expressiveness, and a rich tradition of Catholic and Mediterranean values, which foster both collectivistic tendencies and an emphasis on personal relationships (Triandis, 2018; Hofstede, 2001). At the same time, modern Italian society also exhibits individualistic features, particularly in urban areas and among younger generations. In contrast, South Korea is often characterized as relatively more collectivistic, with emphasis on hierarchical relationships, social harmony, and the prioritization of group needs over individual desires (Buja, 2016; Park and Chesla, 2007). Mental health in Korea remains a stigmatized topic, although growing public awareness has led to gradual changes in attitudes (Seo et al., 2022). These cultural characteristics provide contextual background for the interpretation of the findings but are not directly tested in this study.

**Figure 1:**
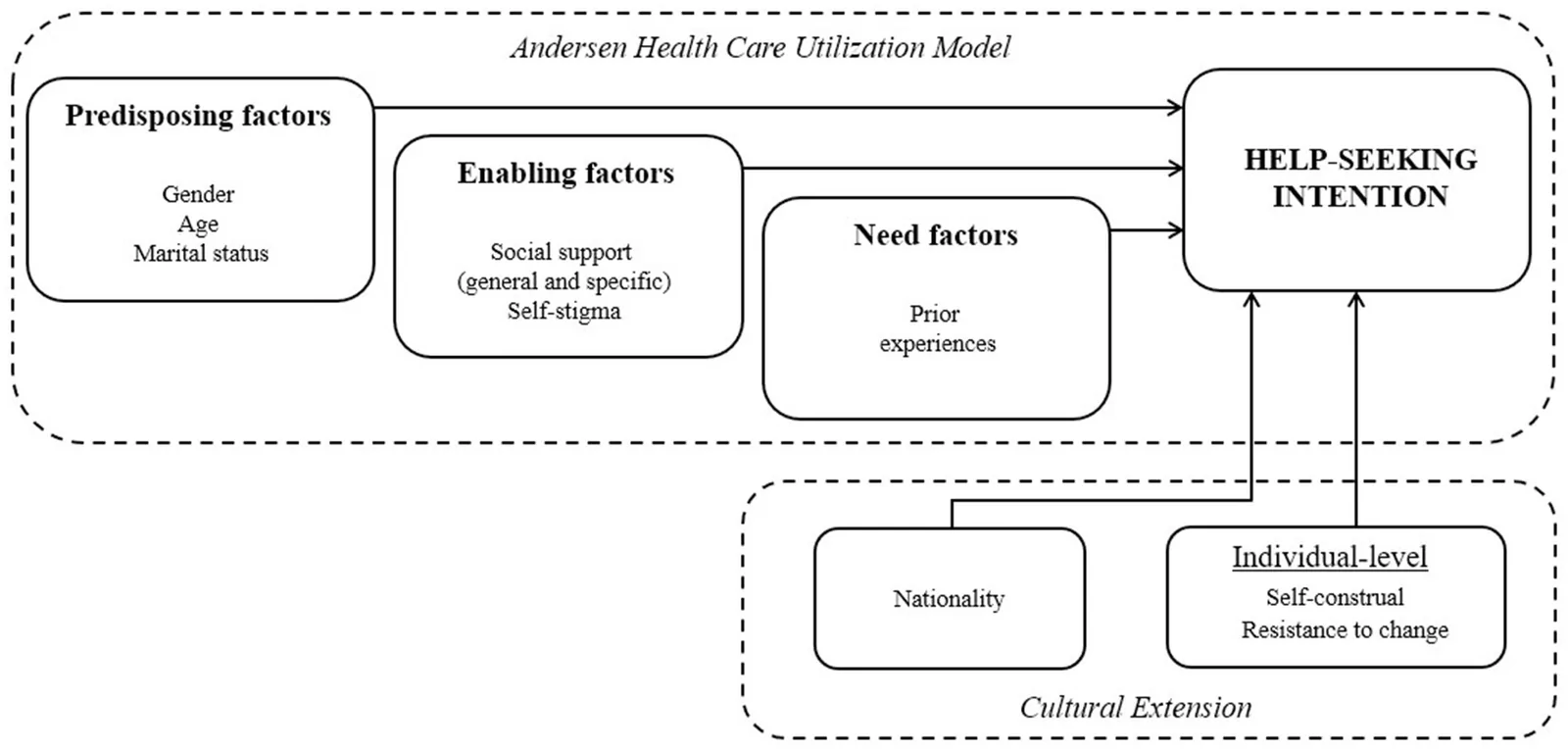
Extended Andersen health care utilization model. The original model is extended with cultural elements comprising one membership indicator (nationality) and two individual-level cultural proxies (self-construal and resistance to change-beliefs).

As illustrated in Figure 1, the study operationalizes the Andersen Model's three components as follows: predisposing characteristics are captured by gender, age, and marital status; enabling factors are represented by two measures of social support (GSS and SMH) and self-stigma; prior experience with mental health services (PEHS) is included as a history-of-care variable and an imperfect proxy for prior need. The model is extended with a cultural block to evaluate whether different conceptualizations of culture contribute additional explanatory value beyond established factors. Because the two conceptualization levels of culture are distinct, the cultural variables are entered into separate steps of a 6-step blockwise hierarchical regression, allowing their independent contributions to be assessed.

### 1.1 Research questions and hypothesis

The present study is guided by the following research question and hypotheses, organized by elements of the Andersen Model. While directional hypotheses are derived from prior literature, given the exploratory nature of this study and its design constraints, they should be interpreted as expectations rather than confirmatory predictions, and findings are interpreted accordingly.

- Predisposing factors: (H1) Female participants will report stronger intentions to seek professional help.
- Enabling factors: (H2) Social support (GSS and SMH) will be significantly associated with intentions to seek professional help when controlling for cultural and demographic variables.
- (H3) Individuals with prior experience seeking professional mental health support will show higher intentions to seek help in the future compared to those without such experience.
- Cultural extension: (RQ1) To what extent does sample membership (nationality) contribute to explaining variations in help-seeking intentions after accounting for demographic and psychological predictors?
- (RQ2, exploratory) How are resistance to change-beliefs and self-construal associated with help seeking intentions within each national sample?

In addition to the hypotheses above, the present study explores gender differences across the study variables as a secondary aim. These analyses are not derived from the Andersen model but are motivated by prior literature suggesting that gender may shape both the predictors and the outcome of interest (Möller-Leimkühler, 2002; Corrigan and Watson, 2002; Vogel et al., 2011).

- (H4) Female participants will report higher rates of social support (both GSS and SMH).
- (H5) Female participants will not differ from male participants in resistance to change-beliefs.
- (RQ3) How will female participants differ in their scores of independent and interdependent self-construal compared to male participants?

## 2 Material and methods

### 2.1 Procedure

This study employed a quantitative cross-sectional design to compare two national university samples of Italian and South Korean university students between the ages of 19 and 35. This research project has been reviewed and accepted by the Università della Svizzera Italiana (USI) Ethical Committee, receiving approval on May 1st, 2023.

The quantitative study involved original primary data collection through a survey distributed either via QR code or a direct link and implemented on the online platform Qualtrics^TM^. For the recruitment process, multiple strategies were used to reach the target population. For the Italian sample, a combination of social media platforms, snowball sampling techniques among university students, and word-of-mouth were employed, no compensation was offered to participants. These approaches facilitated access to a diverse group of participants from various academic faculties and backgrounds. For the Korean sample, data collection was commissioned to a professional survey research company. Eligible panel members aged 19–35 were randomly selected and invited to participate via email; approximately 1,500 invitations were sent. Of these, 320 panel members participated; after excluding respondents who failed the quality check, 150 participants successfully completed the survey. Quota sampling was used to achieve an equal gender distribution across the Korean sample, ensuring comparability with the Italian sample in terms of age range. Participants received $1 as compensation for their time. Several quality-control procedures were implemented to ensure data integrity: duplicate participation screening, exclusion of responses with unusually short completion times, straight-lining detection, and other indicators of insufficient response quality. Only responses passing all quality-control checks were retained. Participants completed the survey online using their mobile device. It should be noted that the two recruitment strategies differ in their structure and are likely to introduce differential response tendencies. This asymmetry is an inherent limitation of this cross-national design and should be considered when interpreting group differences throughout. Participants' eligibility criteria were the ability to effectively understand and complete the questionnaire in the proposed language and an age between 19 and 35 years. The age range of 19–35 was selected to capture the period of emerging and young adulthood, a developmental stage characterized by identity exploration, increasing autonomy and heightened vulnerability to mental health difficulties (Arnett, 2000). The upper age limit was extended beyond the standard threshold of 25 years to 35 years to accommodate older university students and to account for the increased mental health burden documented in young adults during and following the COVID-19 pandemic (Collier Villaume et al., 2023; Krokstad et al., 2022).

The questionnaire consisted of both validated scales and questions created specifically for this research. The questionnaire was back-translated into multiple languages, to assure uniformity between the different versions. As for the administration procedures, after completing the consent form and providing general demographic information (i.e., gender, age), participants proceeded to answer questions on the constructs of interest.

### 2.2 Participants

Demographic characteristics can be found in Table 1. The power calculation was conducted for a two-group mean comparison as a baseline estimate, following Mojaverian et al. (2013). A series of *post hoc* power analysis were conducted in G-Power (Faul et al., 2009), for each step of the primary regression to evaluate the incremental contribution of each block. The analysis resulted in power ranging from 0.78 to 0.997 (Step 2: *N* = 222, predictors = 7, *f* ^2^ = 0.064, α = 0.05; power = 0.78; Step 3: *N* = 222, predictors = 8, *f* ^2^ = 0.111, α = 0.05; power = 0.96; Step 4: *N* = 222, predictors = 9, *f* ^2^ = 0.15, α = 0.05; power = 0.99; Step 5: *N* = 222, predictors = 10, *f* ^2^ = 0.176, α = 0.05; power = 0.996; Step 6: *N* = 222, predictors = 11, *f* ^2^ = 0.19, α = 0.05; power = 0.997). Additionally, the comparison of help-seeking intentions between national groups was not statistically significant (*t*(268.442) = −1.72, *p* = 0.09), which should be considered when interpreting the nationality coefficient that emerges in the regression.

**Table 1:** Demographic characteristics of the Italian and South Korean sample.

Variable	Category	Italian	South Korean	Total sample
Age	M (SD)	26.86 (3.93)	26.39 (5.21)	26.63 (4.61)
	Range	20–35	19–35	19–35
Gender	Female	110	75	185
	Male	40	75	115
	Other	4	0	4
Marital status	Yes	13	19	32
	No	139	127	266
	Missing	2	4	6
*N*		154	150	304

### 2.3 Measures

All the details on scales' means, standard deviations, range, distribution and reliability are presented in Table 2.

**Table 2:** Means, standard deviations, range, reliability and distribution properties of study variables.

Variable	Mean (SD)	Range	Reliability		Distribution	
			*N* items	α	Skewness (SE)	Kurtosis (SE)
GSS	9.99 (2.10)	3–14	3	0.74	−0.42 (0.14)	0.11 (0.28)
SMH	14.82 (4.54)	3–21	3	0.62	−0.62 (0.16)	−0.26 (0.32)
Self-stigma	18.85 (4.76)	6–30	6	0.82	−0.49 (0.14)	−0.06 (0.28)
RC-B	4.28 (0.81)	1.9–6.6	10	0.75	0.24 (0.14)	0.37 (0.28)
Independent SC	4.28 (1.02)	1–6.8	5	0.63	−0.18 (0.14)	−0.23 (0.28)
Interdependent SC	3.82 (0.99)	1.4–6.2	5	0.63	0.03 (0.14)	−0.49 (0.28)
Intention to seek help	2.10 (0.39)	1–2.58	3	0.91	−0.49 (0.14)	−0.53 (0.28)

α = Cronbach's alpha.

#### 2.3.1 Social support

Two measures of social support were used in the questionnaire: one to measure the general perception of one's social circle and the other focused on the perceived support for mental health help-seeking. They are described in detail in the following paragraphs.

##### 2.3.1.1 General social support (GSS)

The validated Oslo Social Support Scale (Kocalevent et al., 2018) was used to measure the level of general social support perceived by the participants. The three items that compose the scale are the following: “*How many people are so close to you that you can count on them if you have great personal problems?”;* “*How much interest and concern do people show in what you do?*”; “*How easy is it to get practical help from your social network if you should need it?*”. Response options ranged on a 4-point Likert scale for the first question (1 = none; 4 = more than five) and a 5-point Likert scale for the second (1 = none; 5 = a lot) and third questions (1 = extremely difficult; 5 = extremely easy). A summative score was calculated with higher scores indicating higher perceived social support. The items showed good reliability (α = 0.74).

##### 2.3.1.2 Support toward mental health (SMH)

This scale was created specifically to capture the perceived support for mental health service utilization by significant people in one's life. The aim was to evaluate the extent to which significant individuals in a person's social circle support seeking psychological help. Participants were asked the following question: “*Think of three influential people in your life and write their role in the space indicated (for example mother, father, sister, friend). For each person listed, indicate how favorable that person might be to seeking help for their mental health using the scale indicated.”* The score ranged from −3 (extremely unfavorable) to +3 (extremely favorable) to provide a real 0 as a cognitive anchor to position their “significant others” in a positive or negative attitude toward help-seeking. In the statistical analysis of the data, the scores were re-coded into a 7-point Likert scale, and the cumulative score was calculated. Higher scores reflect higher support for psychological help-seeking. The variable ranged from 3 to 21 and had an acceptable reliability (α = 0.62).

#### 2.3.2 Self-stigma

To measure self-stigma toward mental illnesses, the 6 items loading significantly on the latent factor “Self-Stigma” were selected from the Stigma and Self-Stigma Scale (Docksey et al., 2022). *If I had a mental disorder, I would feel weak”* is an example of the item presented to the participants, who were asked to answer the questions on a 5-point Likert scale ranging from 1 (strongly disagree) to 5 (strongly agree). The final score was calculated by summing all the points in the six items. Higher scores indicate stronger self-stigma. The scale had good reliability (α = 0.82).

#### 2.3.3 Past experiences with mental help-seeking (PEHS)

Past experiences of help-seeking for mental help problems were investigated with two questions. The items were “*In the last 5 years have you ever been to a mental health professional (psychologist, psychotherapist, psychiatrist, counselor) because of psychological distress?*” and “*If yes, what was the nature of your psychological distress?*”. The response options varied between the two: the first was a dichotomous question (yes = 0; no = 1), while the second had more of a qualitative aim (e.g., anxiety, depression, stress, eating disorder, psychotic disorder).

#### 2.3.4 Self-construal

The short version of the Self-Construal Scale (D'Amico and Scrima, 2016), originally developed by Singelis and Brown (1995), consists of 10 items: five measuring independent self-construal (e.g., “*I do my own thing, regardless of what others think*”) and the remaining five measuring interdependent self-construal (e.g., “*I often have the feeling that my relationships with others are more important than my own accomplishments”*). Participants provided answers on a 7-point Likert scale ranging from 1 (strongly disagree) to 7 (strongly agree). Two scores, one for independent and one for interdependent self-construal have been calculated as the average response to the corresponding items. Higher scores in the two reflect a higher propensity toward independence or interdependence. The reliability was acceptable for both subscales (α = 0.63).

#### 2.3.5 Resistance to change-beliefs (RC-B)

The Resistance to Change-Beliefs (RC-B) scale, developed by White and colleagues in 2020, is composed of two subscales: one that measures the resistance to change and preference for slow changes, the second that measures an individual preference for a traditional way to live. An example of the items for the first subscale is: “Fast or radical changes are unwise and dangerous.”; while an example for the second subscale is: “*Established traditions are the best way to run society”*. Participants expressed their level of agreement with a 7-point Likert scale ranging from 1 (strongly disagree) to 7 (strongly agree). The final score is the average of the items, with higher scores indicating a preference for tradition and gradual changes. The scale had good reliability, with an α = 0.75.

#### 2.3.6 Intention to seek help

The intention to seek help for mental health was measured using the Mental Help Seeking Intention Scale (MHSIS) created and verified by Hammer and Spiker (2018). The three items composing the scale measure different levels of intention for the behavior (e.g., “*If I had a mental health concern, I would try to seek help from a mental health professional.”*) on a 7-point Likert scale, ranging from 1 (extremely unlikely) to 7 (extremely likely). The final score of the scale is calculated as the average of the three items, with higher scores indicating stronger intentions to perform the behavior. The scale had an excellent reliability score (α = 0.91).

### 2.4 Data strategy and statistical analysis

After having assessed the normality distribution of the scales in the study, the outcome variable (i.e., the intention to seek professional help for mental health) was normalized with the square root method. No other variable was normalized or transformed.

To examine whether the multi-item scales functioned equivalently across the Italian and South Korean subsamples, a series of multi-group confirmatory factor analyses were conducted in R using the lavaan package (Rosseel, 2012), with maximum likelihood robust estimation (MLR) and full information maximum likelihood (FIML) for missing data. For each scale, four nested models were tested in sequence—configural, metric, scalar, and partial invariance—and model fit was evaluated using robust fit indices. Decision criteria followed Cheung and Rensvold (2002): ΔCFI ≤ −0.010 and ΔRMSEA ≤ 0.015 indicate acceptable invariance at each transition.

To gather preliminary information on the relationship between the constructs of interest, descriptive statistics and a series of *t*-tests were performed. A Chi-square test was performed to assess the association between the dichotomous variables. Finally, the correlations were tested estimating the Pearson correlation coefficient.

To answer the main research questions and hypotheses, three fixed-sample blockwise hierarchical regression models were estimated, each using listwise deletion on a fixed analytic sample to ensure comparability of changes across steps. Model C constitutes the primary analysis. It uses the subsample of participants with complete data on all variables including SMH (*N* = 222). Predictors were entered in the following blocks: Block 1—predisposing characteristics: gender (dummy variable, male as reference category), age, and marital status (dummy variable, being married as reference category); Block 2—enabling factors and need: self-stigma, general social support (GSS), and prior service use (PEHS; dummy variable, having had prior experience as reference category); Block 3—SMH, entered separately from GSS given its distinct focus on support specifically toward mental health services and its substantially different missing data pattern; Block 4—nationality (dummy variable, Italian as reference category); Block 5—resistance to change-beliefs (RC-B); Block 6—independent and interdependent self-construal. RC-B and self-construal are entered in the final blocks as exploratory variables. Given that measurement invariance was not established for either scale across national groups, their coefficients are not interpreted as reflecting cross-national cultural differences. Model C is treated as the primary analysis because it is the only model that allows H2 to be fully addressed, by including the contribution of SMH. Although the SMH measure has modest reliability and substantial nationality-related missingness, it is a central variable in this study and excluding it entirely from the primary model would prevent any assessment of its role.

Two sensitivity analyses were conducted to isolate SMH's contribution, to allow readers to assess whether results are driven by SMH's inclusion or by sample restriction. Model A uses listwise deletion across all variables except SMH, yielding a larger fixed sample of *N* = 290, and excludes SMH entirely. This model assesses whether results hold in the larger sample when SMH is not included. Model B uses the same restricted sample as Model C (*N* = 222) but excludes SMH, isolating the contribution of SMH by comparing Model B and C coefficients directly. The analyses do not constitute a multilevel model: nationality is entered as a fixed binary predictor, and the coefficient should not be interpreted as estimating between-country variance at a higher level of analysis. Finally, to answer the additional gender analysis (H4, H5 and RQ3), an independent samples *t*-test was computed.

Given the exploratory nature of the study, no formal correction for multiplicity was applied across the analyses. Accordingly, all findings should be treated as preliminary and interpreted with appropriate caution regarding potential inflation of Type I error.

## 3 Results

### 3.1 Measurement invariance testing

The complete measurement invariance tests' results can be found in Table 3. For self-stigma, configural and metric invariance were both supported (configural: CFI =0.966, RMSEA =0.084; metric: ΔCFI = −0.006, *p* = 0.148). Full scalar invariance was not established (ΔCFI = −0.047, *p* < 0.001); however, score tests identified three non-invariant intercepts and three invariant intercepts. A partial scalar invariance model freeing the three non-invariant intercepts did not differ significantly from the metric model (Δχ^2^(2) = 0.311, *p* = 0.856), confirming partial scalar invariance. With three invariant intercepts, cautious latent mean comparisons on self-stigma are defensible (Byrne et al., 1989), though findings should be interpreted provisionally.

**Table 3:** Measurement invariance tests.

Variable (*N* items)	Model	χ^2^ scaled (df)	CFI	TLI	RMSEA [90% CI]	SRMR	ΔCFI	ΔRMSEA
Self stigma (6)								
	Configural	34.31 (18)	0.966	0.943	0.084 [0.056;0.130]	0.038	—	—
	Metric	42.59 (23)	0.960	0.947	0.081 [0.055;0.122]	0.058	−0.006	−0.003
	Scalar	70.29 (28)	0.913	0.907	0.107 [0.084;0.141]	0.076	−0.047^***^	0.026
	Partial scalar^a^	50.54 (25)	0.913	0.907	0.107 [0.076;0.139]	0.076	0.000	0.000
RC-B (10)								
	Configural^b^	262.62 (70)	0.765	0.698	0.142 [0.128;0.163]	0.099	—	—
	Metric	297.55 (79)	0.727	0.689	0.144 [0.131;0.163]	0.129	−0.038^***^	0.002
	Scalar	889.33 (88)	0.045	0.023	0.255 [0.242;0.271]	0.272	−0.683^***^	0.111
Independent SC (5)								
	Configural^b^	84.33 (10)	0.759	0.517	0.207 [0.164;0.251]	0.097	—	—
	Metric	75.24 (14)	0.755	0.649	0.176 [0.140;0.215]	0.105	−0.004	−0.031
	Scalar	98.21 (18)	0.691	0.657	0.174 [0.143;0.209]	0.123	−0.064^**^	−0.002
Interdependent SC (5)								
	Configural	2.99 (10)	1.000	1.123	0.000 [0.000;0.010]	0.018	—	—
	Metric	7.11 (14)	1.000	1.081	0.000 [0.000;0.046]	0.039	0.000	0.000
	Scalar	77.93 (18)	0.560	0.511	0.157 [0.129;0.196]	0.121	−0.440^***^	0.157
Help-seeking intention (3)								
	Configural	Saturated (0)	1.000	1.000	0.000 [—]	0.000	—	—
	Metric	4.46 (2)	0.993	0.980	0.126 [0.052;0.251]	0.050	−0.007	0.126
	Scalar	16.00 (4)	0.976	0.964	0.168 [0.108;0.248]	0.065	−0.017^*^	0.042

All models estimated with MLR; robust fit indices reported throughout. ^*^ *p* < 0.05; ^**^ *p* < 0.01; ^***^ *p* < 0.001. ^a^ Partial scalar model: items 1, 3 and 4 intercepts freed; Δχ^2^(2) = 0.311 vs. metric model, *p* = 0.856. ^b^ Poor configural fit undermines all levels: comparisons not supported.

For resistance to change-beliefs, the single-factor structure showed poor configural fit in both groups (CFI =0.765, RMSEA =0.142). Metric invariance was not supported (ΔCFI = −0.038, *p* < 0.001), and scalar invariance failed completely (ΔCFI = −0.683, *p* < 0.001). These results confirm that the RC-B scale functions differently across Italian and Korean respondents, consistent with concerns about the cross-cultural applicability of a scale developed and validated in a Western context (White et al., 2020).

For self-construal, the two subscales were tested separately. The independent self-construal subscale showed poor configural fit (CFI = 0.759, RMSEA = 0.207), indicating that the five-item factor structure did not hold adequately in either sample, precluding meaningful invariance evaluation. For the interdependent self-construal subscale, configural and metric invariance were fully supported (configural: CFI = 1.000, RMSEA = 0.000; metric: ΔCFI = 0.000, *p* = 0.305). However, scalar invariance failed substantially (ΔCFI = −0.440, *p* < 0.001), with score tests identifying four out of five item intercepts as non-invariant, leaving only one invariant intercept—insufficient to support partial scalar invariance (Byrne et al., 1989). Cross-national comparisons on self-construal are therefore not supported.

For the help-seeking intention scale, the three-item structure was too parsimonious to support a reliable invariance sequence at the configural level, as the just-identified model produced a saturated solution with no degrees of freedom. Metric invariance was tentatively supported (ΔCFI = −0.007), but scalar invariance was not (ΔCFI = −0.017). Cross-national mean comparisons on the outcome measure should therefore be treated as descriptive only.

Overall, full scalar invariance was not established for any of the four scales. These findings indicate that cross-national comparisons in the present study should be treated as exploratory and descriptive rather than as evidence of true differences in underlying latent constructs. All cross-national comparisons are presented with this caveat in mind throughout the Results and Discussion sections.

### 3.2 Preliminary results

The assessment of skewness and kurtosis indicated no problematic deviations from normality in the study variables. Skewness values ranged from −0.62 to 0.24, and kurtosis values from −0.53 to 0.37, all of which fall within the commonly accepted ±1 threshold (Table 2). Consequently, parametric analyses are appropriate for further statistical testing.

Italian participants' ages ranged from 20 to 35, with a mean of 26.86 (SD = 3.93); while Korean participants' ages ranged from 19 to 35, with a mean of 26.39 (SD = 5.21). A *t*-test computed on the variable age revealed that there was no significant difference between the two populations; *t* (302) = 0.89, *p* = 0.37. An overview of the demographic characteristics is provided in Table 1, while the results of the *t*-tests are reported in Table 4.

**Table 4:** *T*-test results on nationality and gender.

Variable	Sample	*N*	M	SD	*t*	df	*p*
Age	IT	154	26.86	3.91	0.89	277.084	0.38
	KR	150	26.39	5.21			
GSS	IT	154	10.54	2.16	4.78	302	< 0.001
	KR	150	9.43	1.88			
	M	115	9.83	2.04	−0.95	298	0.35
	F	185	10.07	2.13			
SMH	IT	151	13.28	4.54	−9.41	222.484	< 0.001
	KR	78	17.79	2.7			
	M	83	15.93	4.38	2.80	224	0.006
	F	143	14.19	4.57			
Self-stigma	IT	154	18.32	4.88	−1.94	302	0.05
	KR	150	19.38	4.59			
RC-B	IT	152	4.41	0.89	2.79	284.227	0.006
	KR	150	4.15	0.69			
	M	115	4.26	0.75	−0.42	296	0.68
	F	183	4.30	0.85			
Independent SC	IT	154	4.19	1.04	−1.59	302	0.11
	KR	150	4.38	1.00			
	M	115	4.49	0.99	2.76	298	0.006
	F	185	4.15	1.03			
Interdependent SC	IT	154	3.82	1.07	−0.09	296.399	0.93
	KR	150	3.83	0.91			
	M	115	3.96	1.01	1.74	298	0.08
	F	185	3.76	0.96			
Intention to seek help	IT	150	2.08	0.45	−1.72	268.442	0.09
	KR	150	2.15	0.32			
	M	115	2.06	0.37	−1.88	295	0.06
	F	182	2.15	0.39			

IT, Italian Sample; KR, South-Korean Sample. M, males; F, females.

Regarding past experiences with mental health, 50% of Italian participants revealed they had been to a mental health professional in the past 5 years (*N* = 77), and one participant decided not to answer. Anxiety and depression, followed by eating disorders, were the most common causes for seeking psychological help. On the other hand, 12.7% of the Korean population reported to have been to a professional for their mental health in the last 5 years (*N* = 19), and four people did not respond to this item. The main reasons listed for their distress were stress, anxiety, and depression. To test the differences in the distribution of the dichotomous variable of past experiences (PEHS) on nationality, a Chi-square test was performed. A chi-square test of independence was performed to evaluate the relationship between past experiences (PEHS) and nationality. The relationship between these variables was significant, χ^2^ (1, *N* = 299) = 47.72, *p* = < 0.001. Italians were more likely to have had past experiences with mental health professionals compared to South Koreans.

Multiple independent sample *t*-tests were performed to analyze the different distributions of the scores between the two populations, the results can be found in Table 4. The participants coming from two different nationalities differed in a number of measures. Italians had higher observed scores in perceived general social support (GSS) and resistance to change-belief (RC-B) but lower observed scores in support toward mental health (SMH), and self-stigma.

Finally, the association between the continuous study variables was tested and the results are presented in Table 5. Age was only significantly associated with general social support (*r* = −0.15, *p* < 0.05). General social support was associated with independent self-construal (*r* = 0.20, *p* < 0.01) and self-stigma (*r* = −0.18, *p* < 0.01). Support toward mental health was correlated with independent self-construal (*r* = 0.21, *p* < 0.01) and the Resistance to Change-Belief scale (*r* = −0.16, *p* < 0.05). Interdependent self-construal was correlated with self-stigma (*r* = 0.24, *p* < 0.01) and lastly the resistance to change-belief scale was associated with the outcome variable, the intention to seek professional help (*r* = 0.15, *p* < 0.01).

**Table 5:** Correlations between continuous study variables.

Variables	1.	2.	3.	4.	5.	6.	7.	8.
1. Age	–							
2. GSS	−0.15^*^	–						
3. SMH	−0.09	0.08	–					
4. Self-stigma	0.06	−0.18^**^	−0.04	–				
5. RC-B	0.03	0.09	−0.16^*^	0.05	–			
6. Independent SC	−0.03	0.20^**^	0.21^**^	−0.07	0.03	–		
7. Interdependent SC	0.02	0.06	−0.04	0.24^**^	0.10	0.05	–	
8. Intention to seek help	0.06	0.10	0.11	−0.09	0.15^**^	0.12	0.01	–

^*^ *p* value < 0.05; ^**^ *p* value < 0.01.

An independent-samples *t*-test was conducted to compare intention to seek help between the two samples (Italians and South Koreans). Levene's test indicated unequal variances, *F*(1,298) = 32.24, *p* < 0.001. The analysis showed no statistically significant difference between the groups, *t*(268.44) = −1.72, *p* = 0.087. The mean difference was −0.08 (95% CI [−0.166, 0.011]). This observed difference is reported as descriptive only; given that scalar measurement invariance was not established for the outcome measure. It should also be noted that the observed pattern of group differences throughout this study may partly reflect differences in recruitment methods rather than cultural factors alone.

Due to the high missingness of data on the SMH scale, an independent samples *t*-test was conducted to examine differences between responders and non-responders. The two groups did not differ significantly in age (*t*(108.004) = 1.230, *p* = 0.222, *d* = 0.18, 95% CI [−0.511, 2.179]) or gender composition (*t*(298) = −0.999, *p* = 0.319, *d* = −0.13, 95% CI [−0.194,0.063]). They also did not differ significantly in help-seeking intentions (*t*(298) = 0.226, *p* = 0.821, *d* = 0.03, 95% CI [−0.092,0.116]). Critically, they differed in terms of nationality, with South Koreans having significantly more non-responders (*t*(286.513) = 15.971, *p* < 0.001, *d* = 1.461, 95% CI [0.543,0.696]). This is the reason why a set of sensitivity analyses were conducted to assess the robustness of results.

### 3.3 Sensitivity analyses

To assess the robustness of the primary findings, two sensitivity analyses were conducted using alternative fixed-sample models. Model A (*N* = 290; Table 6) excluded SMH entirely and used a larger listwise sample including participants without complete SMH data. Model B (*N* = 222; Table 7) used the same restricted sample as Model C but excluded SMH, allowing direct isolation of SMH's contribution by comparison with Model C.

**Table 6:** Regression coefficients of study variables on Intention to seek help (Model A: excluding SMH and applying listwise deletion).

Variable	Step 1			Step 2			Step 3			Step 4			Step 5		
	B	β	SE	B	β	SE	B	β	SE	B	β	SE	B	β	SE
Constant	1.69^**^ [1.32; 2.07]		0.19	1.85^**^ [1.35; 2.36]		0.26	1.63^**^ [1.13; 2.14]		0.26	1.35^**^ [0.82; 1.89]		0.27	1.22^**^ [0.67; 1.78]		0.28
Gender^a^	0.08 [−0.01;0.17]	0.10	0.05	0.05 [−0.04;0.14]	0.07	0.05	0.09^*^ [0.003;0.18]	0.12	0.05	0.09^*^ [0.005;0.18]	0.12	0.05	0.11^*^ [0.02;0.19]	0.13	0.05
Age	0.01 [−0.001;0.02]	0.11	0.01	0.01 [−0.002;0.02]	0.10	0.01	0.01^*^ [0.00;0.02]	0.13	0.01	0.01 [0.00;0.02]	0.12	0.01	0.01 [0.00;0.02]	0.12	0.01
Marital status^b^	0.14 [−0.02;0.30]	0.11	0.08	0.08 [−0.08;0.24]	0.07	0.08	0.08 [−0.07;0.24]	0.07	0.08	0.08 [−0.07;0.24]	0.07	0.08	0.09 [−0.06;0.24]	0.08	0.08
GSS				0.02 [−0.01;0.04]	0.08	0.01	0.03^*^ [0.005;0.05]	0.14	0.01	0.02^*^ [0.003;0.05]	0.13	0.01	0.02 [−0.002;0.04]	0.11	0.01
Self-stigma				−0.01 [−0.02;0.004]	−0.07	0.01	−0.01 [−0.02;0.002]	−0.09	0.01	−0.01 [−0.02;0.001]	−0.10	0.01	−0.01 [−0.02;0.002]	−0.09	0.01
PEHS^c^				−0.16^**^ [−0.26; −0.07]	−0.20	0.05	−0.24^**^ [−0.34; −0.14]	−0.29	0.05	−0.24^**^ [−0.33; −0.14]	−0.28	0.05	−0.24^**^ [−0.34; −0.15]	−0.29	0.05
Nationality^d^							0.21^**^ [0.11;0.31]	0.27	0.05	0.23^**^ [0.13;0.32]	0.29	0.05	0.22^**^ [0.12;0.31]	0.28	0.05
RC-B										0.07^**^ [0.02;0.13]	0.16	0.03	0.07^**^ [0.02;0.12]	0.15	0.03
Independent SC													0.05^*^ [0.003;0.09]	0.12	0.02
Interdependent SC													−0.01 [−0.05;0.04]	−0.02	0.02
*R* ^2^	0.02			0.06			0.11			0.13			0.14		
Δ*R*^2^	0.01			0.05^**^			0.06^***^			0.02^*^			0.01		

Missing data were handled using listwise deletion; *N* = 290; Dependent variable: Intention to seek help. Reference categories for binary variables: ª Male = 0; ^b^ Being married = 0; ^c^ Having previous experience = 0; ^d^ Italian = 0. ^*^ *p* value < 0.05; ^**^ *p* value < 0.01; ^***^ *p* value < 0.001.

**Table 7:** Regression coefficients on Intention to seek help (Model B: participants with SMH data and applying listwise deletion).

Variable	Step 1			Step 2			Step 3			Step 4			Step 5		
	B	β	SE	B	β	SE	B	β	SE	B	β	SE	B	β	SE
Constant	1.68^**^ [1.22; 2.14]		0.23	1.95^**^ [1.32; 2.59]		0.32	1.83^**^ [1.22; 2.45]		0.31	1.59^**^ [0.94; 2.24]		0.33	1.40^**^ [0.72; 2.07]		0.34
Gender^a^	0.07 [−0.03;0.18]	0.09	0.06	0.05 [−0.06;0.16]	0.06	0.05	0.10 [−0.002;0.21]	0.13	0.05	0.10 [−0.01;0.20]	0.12	0.05	0.11^*^ [0.005;0.22]	0.14	0.05
Age	0.01 [−0.004;0.02]	0.11	0.01	0.01 [−0.005;0.02]	0.09	0.01	0.01 [−0.002;0.02]	0.12	0.01	0.01 [−0.003;0.02]	0.11	0.01	0.01 [−0.003;0.02]	0.11	0.01
Marital status^b^	0.15 [−0.06;0.35]	0.11	0.10	0.08 [−0.12;0.28]	0.06	0.10	0.06 [−0.13;0.26]	0.05	0.10	0.05 [−0.14;0.24]	0.04	0.10	0.07 [−0.13;0.26]	0.05	0.10
GSS				0.01 [−0.01;0.04]	0.06	0.01	0.02 [−0.01;0.04]	0.08	0.01	0.02 [−0.01;0.04]	0.07	0.01	0.01 [−0.01;0.04]	0.06	0.01
Self-stigma				−0.01 [−0.02;0.002]	−0.10	0.01	−0.01^*^ [−0.02;0.00]	−0.13	0.01	−0.01^*^ [−0.02;0.000]	−0.13	0.01	−0.01 [−0.02;0.003]	−0.10	0.01
PEHS^c^				−0.18^**^ [−0.29; −0.07]	−0.25	0.06	−0.26^**^ [−0.38; −0.15]	−0.32	0.06	−0.26^**^ [−0.37; −0.15]	−0.31	0.06	−0.27^**^ [−0.38; −0.16]	−0.33	0.06
Nationality^d^							0.25^**^ [0.13;0.37]	0.30	0.06	0.26^**^ [0.15;0.38]	0.32	0.06	0.25^**^ [0.13;0.37]	0.30	0.06
RC-B										0.06^*^ [0.003;0.12]	0.13	0.03	0.06^*^ [0.003;0.12]	0.13	0.03
Independent SC													0.06^*^ [0.006;0.11]	0.14	0.03
Interdependent SC													−0.01 [−0.07;0.04]	−0.04	0.03
*R* ^2^	0.01			0.06			0.13			0.14			0.15		
Δ*R*^2^	0.01			0.06^**^			0.07^***^			0.02^*^			0.02		

Missing data were handled using listwise deletion; *N* = 222; Dependent variable: Intention to seek help. Reference categories for binary variables: ª Male = 0; ^b^ Being married = 0; ^c^ Having previous experience = 0; ^d^ Italian = 0. ^*^ *p* value < 0.05; ^**^ *p* value < 0.01; ^***^ *p* value < 0.001.

Across all three models, prior service use retained the same direction and significance throughout (Model A Step 5: *B* = −0.24, *p* < 0.001; Model B Step 5: *B* = −0.27, *p* < 0.001; Model C Step 6: *B* = −0.28, *p* < 0.001), confirming this as the most robust finding in the study. Nationality remained significantly associated with help-seeking intentions in all models (Model A: *B* = 0.22, *p* < 0.001; Model B: *B* = 0.25, *p* < 0.001; Model C: *B* = 0.21, *p* = 0.001), though its coefficient was somewhat larger in Models A and B than in Model C, consistent with SMH partially accounting for the sample-membership variation captured by nationality once included in Model C. Resistance to change-beliefs was significant in Models A and C but borderline in Model B (Model A Step 4: *B* = 0.07, *p* = 0.006; Model B Step 5: *B* = 0.06, *p* = 0.039; Model C Step 6: *B* = 0.07, *p* = 0.025), indicating some sensitivity to sample composition. General social support (GSS) was not significant in any step of any model. Independent self-construal showed borderline significance in the final step of all three models (Model A: *p* = 0.035; Model B: *p* = 0.030; Model C: *p* = 0.047); however, given the measurement invariance concerns identified for this scale, this finding should not be treated as substantive. Overall, the sensitivity analyses support the robustness of the primary findings for prior service use and nationality, while highlighting that resistance to change-belief and independent self-construal should be treated with additional caution given their sensitivity to sample composition and measurement non-equivalence across groups.

### 3.4 Hypothesis testing

The results reported below refer to Model C (*N* = 222), the primary fixed-sample analysis including all predictors (Table 8). Contrary to H1, female participants did not differ significantly from male participants in their intention to seek professional help in the independent samples *t*-test, conducted on the available sample (*t*(295) = −1.88, *p* = 0.06). Gender emerged as a significant predictor in the regression in Steps 4 and 6 (Step 4: *B* = 0.11, *p* = 0.045; Step 6: *B* = 0.11, *p* = 0.036), underscoring the influence previously recognized by research.

**Table 8:** Regression coefficients of study variables (Model C: primary analysis; with all variables and listwise deletion).

Variable	Step 1			Step 2			Step 3			Step 4			Step 5			Step 6		
	B	β	SE	B	β	SE	B	β	SE	B	β	SE	B	β	SE	B	β	SE
Constant	1.68^**^ [1.22; 2.14]		0.23	1.95^**^ [1.32; 2.59]		0.32	1.65^**^ [0.99; 2.30]		0.33	1.68^**^ [1.04; 2.32]		0.32	1.39^**^ [0.72; 2.07]		0.34	1.24^**^ [0.54; 1.94]		0.36
Gender^a^	0.07 [−0.03;0.18]	0.09	0.06	0.05 [−0.06;0.16]	0.06	0.05	0.07 [−0.03;0.18]	0.09	0.05	0.11^*^ [0.002;0.21]	0.13	0.05	0.10 [−0.004;0.21]	0.12	0.05	0.11^*^ [0.008;0.22]	0.14	0.05
Age	0.01 [−0.004;0.02]	0.11	0.01	0.01 [−0.005;0.02]	0.09	0.01	0.01 [−0.003;0.02]	0.10	0.01	0.01 [−0.002;0.02]	0.12	0.01	0.01 [−0.002;0.02]	0.11	0.01	0.01 [−0.002;0.02]	0.11	0.01
Marital status^b^	0.15 [−0.06;0.35]	0.11	0.10	0.08 [−0.12;0.28]	0.06	0.10	0.09 [−0.10;0.29]	0.07	0.10	0.08 [−0.12;0.27]	0.05	0.10	0.07 [−0.13;0.26]	0.05	0.10	0.08 [−0.12;0.27]	0.06	0.10
GSS				0.01 [−0.01;0.04]	0.06	0.01	0.01 [−0.02;0.04]	0.05	0.01	0.01 [−0.01;0.04]	0.07	0.01	0.01 [−0.01;0.04]	0.06	0.01	0.01 [−0.02;0.04]	0.05	0.01
Self-stigma				−0.01 [−0.02;0.002]	−0.10	0.01	−0.01 [−0.02;0.003]	−0.09	0.01	−0.01 [−0.02;0.001]	−0.12	0.01	−0.01 [−0.02;0.001]	−0.12	0.01	−0.01 [−0.02;0.003]	−0.09	0.01
PEHS^c^				−0.18^**^ [−0.29; −0.07]	−0.21	0.06	−0.22^**^ [−0.33; −0.11]	−0.27	0.06	−0.27^**^ [−0.39; −0.16]	−0.33	0.06	−0.27^**^ [−0.38; −0.16]	−0.33	0.06	−0.28^**^ [−0.39; −0.17]	−0.34	0.06
SMH							0.02^**^ [0.007;0.030]	0.22	0.01	0.01 [−0.002;0.023]	0.12	0.01	0.01 [0.000;0.025]	0.14	0.01	0.01 [−0.002;0.023]	0.12	0.01
Nationality^d^										0.21^**^ [0.08;0.34]	0.25	0.06	0.21^**^ [0.09;0.34]	0.26	0.06	0.21^**^ [0.08;0.33]	0.25	0.06
RC-B													0.07^*^ [0.010;0.127]	0.15	0.03	0.07^*^ [0.009;0.126]	0.14	0.03
Independent SC																0.05^*^ [0.001;0.10]	0.13	0.03
Interdependent SC																−0.01 [−0.06;0.04]	−0.03	0.03
*R* ^2^	0.01			0.06			0.10			0.13			0.15			0.16		
Δ*R*^2^	0.01			0.06^**^			0.04^**^			0.04^***^			0.02^*^			0.02		

Missing data were handled using listwise deletion; *N* = 222; Dependent variable: Intention to seek help.

Reference categories for binary variables: ª Male = 0; ^b^ Being married = 0; ^c^ Having previous experience = 0; ^d^ Italian = 0.

^*^ *p* value < 0.05; ^**^ *p* value < 0.01; ^***^ *p* value < 0.001.

Results were partially consistent with H2. GSS was not significantly associated with help-seeking intentions at any step (Step 6: *B* = 0.01, *p* = 0.457). SMH was positively and significantly associated with intentions when first entered in Step 3 (*B* = 0.02, *p* = 0.002, 95% CI [0.007; 0.030]), but lost significance with the addition of nationality in Step 4 (*B* = 0.01, *p* = 0.098) and remained non-significant in subsequent steps (Step 6: *B* = 0.01, *p* = 0.094). This pattern suggests that the association between SMH and help-seeking intentions may be partially accounted for by the membership variation captured by nationality.

Consistent with H3, participants without prior experience with mental health services reported lower help-seeking intentions compared to those with prior experience (Step 6: *B* = −0.28, *p* < 0.001, 95% CI [−0.394; −0.170]), even after controlling for all other variables. This was the most stable and robust finding across all steps and all three models.

RQ1 can be answered by looking at Steps 4, 5, and 6 of the hierarchical regression in Table 8. Nationality showed the largest standardized association and remained significant across Steps 4 through 6 (Step 6: *B* = 0.21, β = 0.251, *p* = 0.001, 95% CI [0.084; 0.334]). Given that the two national samples were recruited through structurally different methods, this association should be interpreted cautiously. Furthermore, since scalar measurement invariance was not established for the outcome measure, the observed nationality difference should be treated as a descriptive difference in observed scores rather than as evidence of a true difference in underlying help-seeking intentions.

Regarding RQ2, resistance to change-beliefs was positively associated with help-seeking intentions (Step 6: *B* = 0.07, β = 0.143, *p* = 0.025, 95% CI [0.009; 0.126]). The possibility of a reverse-coding error was ruled out through verification of the scale's scoring direction. Independent self-construal showed a borderline association with help-seeking intentions in the final step (*B* = 0.05, β = 0.131, *p* = 0.047, 95% CI [0.001; 0.102]), while interdependent self-construal was not significant (*B* = −0.01, β = −0.031, *p* = 0.634). Given the measurement invariance concerns identified for both of these scales, the findings should not be interpreted as substantive and are reported for completeness and exploratory purposes only.

Gender differences were examined through a series of independent samples *t*-tests. Regarding H4, female participants did not differ significantly from male participants in general social support (*t*(298) = −0.95, *p* = 0.35, *d* = −0.11) but reported lower support from significant others toward mental health practices (SMH; *t*(224) = 2.80, *p* = 0.006, *d* = 0.39). No significant gender difference was found in resistance to change-beliefs (*t*(296) = −0.42, *p* = 0.68, *d* = −0.05), consistent with H5, though the absence of a significant effect does not confirm equivalence and a small undetected difference cannot be excluded. Regarding RQ3, female participants scored significantly lower on the independent self-construal scale (*t*(298) = 2.76, *p* = 0.006, *d* = 0.33) but did not differ significantly from male participants on interdependent self-construal (*t*(298) = 1.74, *p* = 0.083, *d* = 0.21).

## 4 Discussion

The present study explored the correlates of observed mental health help-seeking intention scores in two independently recruited national student samples by integrating the Andersen Health Care Utilization Model with additional exploratory variables. The following discussion addresses each major finding in turn, with the interpretive caution appropriate to an exploratory cross-national design.

### 4.1 Predisposing factor: gender

Gender emerged as a statistically significant variable in Step 4 and 6 of the regression model (Table 8), with female participants showing higher help-seeking observed intention scores. This is consistent with the broader literature documenting women's greater likelihood of seeking professional mental health support relative to men (Abdelmonaem et al., 2024; Möller-Leimkühler, 2002; Qiu et al., 2024). However, several of the gender-specific associations tested in this study were not aligned with previous literature, and the pattern of results is more nuanced than the general trend suggests.

Female participants did not report higher general social support, though they did report lower support from significant others for mental health service use (SMH), a finding that runs counter to the expected direction and which remains difficult to interpret without further data (H4). In terms of H5, the analyses were consistent in finding no gender difference in resistance to change-beliefs, suggesting that conformity beliefs in these samples are not differentially gendered. While this finding suggests the absence of a strong gender association with traditionalism in these samples, it should not be taken as confirmation of full equivalence, and the possibility of a small undetected difference cannot be excluded. Regarding self-construal (RQ3), women scored significantly lower on independent self-construal but did not differ from men on interdependent self-construal, partially consistent with prior research suggesting that women more often endorse relational rather than agentic self-definitions (Markus and Kitayama, 1991; Corrigan and Watson, 2002).

It should also be noted that the gender composition of the two national samples was unequal: 71% of Italian participants identified as female, compared to 50% of Koreans. This difference is partly a methodological artifact rather than a purely cultural one: the Korean sample was actively balanced to an equal gender distribution through quota sampling by design, while the Italian sample was recruited with no gender quota. Future studies should aim for more balanced sampling across gender and nationality to allow cleaner interpretation of main effects and interactions.

### 4.2 Enabling factor: social support

The two measures of social support produced different patterns, both across nationalities and in their relationship with the outcome variable. Italian participants reported significantly higher general social support (GSS), while Korean participants reported higher support specifically oriented toward mental health service utilization (SMH). In the regression, GSS was not significantly associated with help-seeking intentions at any step, while SMH was significant when first entered (*B* = 0.02, *p* = 0.002) but lost its significance with the addition of nationality (*B* = 0.011, *p* = 0.098) suggesting that its effect may be partially absorbed or confounded by this variable. The divergence between the two types of social support is theoretically meaningful. It suggests that feeling generally supported by one's social network may not be sufficient to drive help-seeking intentions; rather, what seems to matter is whether those specific social ties endorse and normalize professional psychological help. This distinction aligns with theoretical arguments about the role of injunctive norms—the perceived approval of significant others for a specific behavior—as distinct from broader social embeddedness (Vogel and Wei, 2005; Pescosolido and Boyer, 2010). The Italian pattern, in which robust general support coexists with lower endorsement of professional help-seeking, may partly point to why Italians' observed mean help-seeking intentions were slightly lower: strong informal networks may function as a substitute for professional services, reducing perceived necessity rather than facilitating access.

It is important to note, however, that the Korean SMH data were substantially incomplete: only 78 of the 150 Korean participants contributed to the SMH analyses, compared to 151 of 154 Italians. This imbalance represents a meaningful gap. The lower Korean completion rate on this scale may reflect discomfort with evaluating significant others' attitudes toward mental health, or it may be an artifact of the mobile survey format used for recruitment. Furthermore, the low reliability of the scale warrants attention in the interpretation of the results.

### 4.3 Prior experiences

Consistent with H3, participants without prior experience with mental health services reported lower help-seeking intentions compared to those with prior experience (*B* = −0.28, *p* < 0.01). This aligns with the premise that previous service lowers barriers and normalizes professional help-seeking. Individuals who have sought help before may have developed greater familiarity with the therapeutic process. This finding is also consistent with literature suggesting that prior contact with professional services reduces barriers and normalizes future help-seeking behavior (Elshaikh et al., 2023). Although prior service use is not a direct operationalization of the Andersen model's need component it may still reflect a relevant pathway.

It is worth noting that prior experiences rates differed substantially between the two groups: 50% of Italians reported prior experience with mental health services, compared to only 12.7% of Koreans. This compositional difference may partly contribute to the nationality effect observed in the regression, given that nationality and prior experience were entered in separate blocks of the regression. These effects, while statistically separable, may not be fully independent in the real-world context of these two samples.

### 4.4 Cultural extension: nationality

South Korean participants reported marginally higher observed scores on the intention to seek professional mental health support. At the same time, nationality had the largest standardized regression coefficient in the final blockwise hierarchical regression model (*B* = 0.21, β = 0.251, *p* = 0.001, 95% CI [0.084; 0.334]). In this case, nationality functions as a sample-membership indicator that distinguished two groups recruited through structurally different procedures. Since scalar measurement invariance was not established for the outcome measure, this observed difference should be treated as a descriptive difference in observed scores between the two independently recruited samples.

Several explanations are possible for this result, including elevated perceived normativity of help-seeking (Hwang et al., 2008; Kim et al., 2025), differences in public discourse (Kim et al., 2025; Rim et al., 2023), informal support structures (Galán-Vera et al., 2025; Luciano et al., 2012) and academic contexts (Jarvis et al., 2020). Because none of these mechanisms were measured directly, they remain speculative and should be evaluated in future research.

A related consideration is that the Korean recruitment method differed from the Italian one: the Korean sample was collected through an external private company using mobile surveys, while the Italian sample relied on social media and snowball sampling. Although both methods were designed to reach university students, the structural differences in data collection may have introduced differential response tendencies that are difficult to disentangle from cultural effects.

### 4.5 Cultural extension: individual-level

The positive association between resistance to change-beliefs (RC-B) and help-seeking intentions (RQ2; *B* = 0.07, β = 0.143, *p* = 0.025, 95% CI [0.009; 0.126]) should be interpreted with particular caution given that measurement invariance was not established. Past research linked conservative worldviews to stigma, self-reliance norms, and reduced openness to external interventions (Obasi and Leong, 2009; Boski et al., 2004). The data suggest the opposite. A plausible explanation, that requires further empirical investigation, lies in the nature of what resistance to change-beliefs may mean within these specific cultural contexts. For individuals embedded in cultures that emphasize stable social roles, family duty, and community wellbeing, seeking professional help may not represent a departure from tradition but rather a fulfillment of one's obligation to maintain functional participation in family and social life (Yang and Kleinman, 2008; Taylor et al., 2004). In this reading, help-seeking could be a responsible response to psychological distress that ensures one can continue to meet relational obligations. This interpretation aligns with the collectivistic rationale for help-seeking described in the introduction, in which the health of the individual is understood as instrumental to the health of the group. However, due to the limitations discussed above, this interpretation is highly speculative and requires direct empirical testing in future research with psychometrically equivalent measures.

As exploratory findings, self-construal associations with help-seeking intentions were largely non-significant. Independent self-construal showed a borderline association in the final step of the primary model (*B* = 0.05, *p* = 0.047), while interdependent self-construal was not significant (*B* = −0.01, *p* = 0.634). Self-construal has been widely used as a shorthand for cultural orientation, particularly in distinguishing individualistic from collectivistic tendencies. However, self-construal is an individual-difference variable: it captures how a specific person relates to others, not the socio-structural or normative context in which they are embedded. This is consistent with growing critiques of the individualism-collectivism paradigm, which argue that these categories are too broad and internally diverse to generate reliable behavioral predictions, particularly when applied to specific, context-dependent behaviors like help-seeking (Levesque and Li, 2014; Omori, 2007). It is plausible that the decision to seek professional psychological help is shaped more by institutional availability and socially shared norms on distress and disclosure than by an individual's generalized sense of independence or interdependence.

### 4.6 Limitations

Some limitations need to be acknowledged. The cross-sectional design precludes causal inference. The constructs examined are treated as antecedents of help-seeking intentions, but the directionality of some of these relationships may be more complex.

The study relied on sampling of university students, limiting generalizability to the broader adult populations. Furthermore, the two national samples were recruited using different methods, which may have introduced differential response sets, social desirability pressures, or sampling biases that are difficult to isolate from genuine cultural differences. Although these concerns cannot be fully resolved *post hoc*, they are worth flagging, and future cross-national studies should aim for greater methodological symmetry in recruitment. In addition, future research would benefit from including a validated measure of social desirability as a control variable to better disentangle genuine attitudinal differences from response tendencies.

The SMH scale, developed specifically for this study, had modest internal reliability (α = 0.62) and, critically, was completed by only 78 of the 150 Korean participants. This substantial rate of missing data on a key variable not observed in the Italian sample could reflect survey fatigue or cultural discomfort with the item content. The SMH measure itself requires further validation across cultural contexts before being used as a primary variable in cross-national comparisons.

A further limitation concerns measurement comparability across the two national groups. Formal measurement invariance testing was conducted using multi-group confirmatory factor analysis. The findings suggest that cross-national mean comparison and regression coefficients involving these scales should be treated as descriptive and provisional, and cross-national differences may partly reflect measurement non-equivalence rather than true differences in the underlying constructs.

The study's outcome variable captures a crucial but proximal step in the help-seeking process. Intentions do not always translate into behavior, and the conditions under which this translation occurs or fails may themselves be culturally patterned. Future research should move toward measuring actual help-seeking behavior, or at minimum integrating measures of implementation intentions and perceived behavioral control, to more fully map the pathway from attitude to action.

Finally, while the study compares two national samples, both Italy and South Korea contain substantial internal cultural diversity. These within-country differences may be as consequential as between-country differences for help-seeking behavior, and future work should move toward more granular cultural sampling strategies.

### 4.7 Recommendations for future research

The exploratory findings presented so far open several productive avenues for future investigation. First, the present study highlights the importance of establishing measurement equivalence before drawing cross-national conclusions. Future research should prioritize the development and cross-cultural validation of measures specifically adapted for use in Western and East Asian populations before attempting to identify cultural mechanisms underlying help-seeking differences.

Second, the positive relationship between resistance to change-beliefs and help-seeking intentions deserves further investigation. Future studies should examine whether this effect replicates across other cultural contexts, and whether it is moderated by the type of help-seeking (informal vs. professional, preventive vs. crisis-driven) or by the framing of professional services within a given community.

Third, given the limitations of the SMH scale identified above, future research should invest in developing and validating culturally adapted measures of social support specifically oriented toward mental health service use.

Finally, incorporating social desirability scales, behavioral measures of actual service use, and longitudinal designs would significantly strengthen the causal and predictive conclusions. The field will advance most quickly through designs that track intention, access, and experience across time and national contexts.

## Data Availability

The raw data supporting the conclusions of this article will be made available by the authors, without undue reservation.

## References

[B1] AbdelmonaemY. M. M. OsmanM. A. KarimN. A. H. A. (2024). Mental health stigma and internship nursing students' attitudes toward seeking professional psychological help: a cross-sectional study. BMC Nurs. 23:275. doi: 10.1186/s12912-024-01910-338658957 PMC11044461

[B2] AlkhawaldehA. Al BashtawyM. RayanA. AbdalrahimA. MusaA. EshahN. . (2023). Application and use of Andersen's behavioral model as theoretical framework: a systematic literature review from 2012–2021. Iran. J. Public Health 52, 1346–1354. doi: 10.18502/ijph.v52i7.1323637593505 PMC10430393

[B3] Andersen R. Newman J. F. (1973). Societal and individual determinants of medical care utilization in the United States. Milbank Mem. Fund Q. Health Soc. 51, 95–124. doi: 10.2307/3349613 4198894

[B4] Andersen R. M. (1995). Revisiting the behavioral model and access to medical care: does it matter? J. Health Soc. Behav. 36, 1–10. doi: 10.2307/2137284 7738325

[B5] Arnett J. J. (2000). Emerging adulthood: a theory of development from the late teens through the twenties. Am. Psychol. 55, 469–480. doi: 10.1037/0003-066X.55.5.469 10842426

[B6] Babitsch B. Gohl D. von Lengerke T. (2012). Re-revisiting Andersen's behavioral model of health services use: a systematic review of studies from 1998–2011. Psychosoc. Med. 9:Doc11. doi: 10.3205/psm000089 PMC3488807 23133505

[B7] Boski P. Strus K. Tlaga E. (2004). “Cultural identity, existential anxiety and traditionalism,” in Ongoing Themes in Psychology and Culture: Proceedings from the 16th International Congress of the International Association for Cross-Cultural Psychology, eds. B. N. Setiadi, A. Supratiknya, W. J. Lonner, and Y. H. Poortinga (International Association for Cross-Cultural Psychology) (IACCP)), 457–474. doi: 10.4087/YWUT5806

[B8] Buja E. (2016). Hofstede's dimensions of national cultures revisited: a case study of South Korea's culture. Acta Univ. Sapientiae Philol. 8, 169–182. doi: 10.1515/ausp-2016-0012

[B9] Byrne B. M. Shavelson R. J. Muthén B. (1989). Testing for the equivalence of factor covariance and mean structures: the issue of partial measurement invariance. Psychol. Bull. 106, 456–466. doi: 10.1037/0033-2909.105.3.456

[B10] Cheung G. W. Rensvold R. B. (2002). Evaluating goodness-of-fit indexes for testing measurement invariance. Struct. Equ. Model. 9, 233–255. doi: 10.1207/S15328007SEM0902_5

[B11] Collier VillaumeS. ChenS. AdamE. K. (2023). Age disparities in prevalence of anxiety and depression among US adults during the COVID-19 pandemic. JAMA Netw. Open 6:e2345073. doi: 10.1001/jamanetworkopen.2023.4507338032641 PMC10690464

[B12] CorriganP. W. RaoD. (2012). On the self-stigma of mental illness: stages, disclosure, and strategies for change. Can. J. Psychiatry 57, 464–469. doi: 10.1177/07067437120570080422854028 PMC3610943

[B13] CorriganP. W. WatsonA. C. (2002). Understanding the impact of stigma on people with mental illness. World Psychiatry 1, 16–20.16946807 PMC1489832

[B14] D'Amico A. Scrima F. (2016). The Italian validation of Singelis's Self-Construal Scale (SCS): a short 10-item version shows improved psychometric properties. Curr. Psychol. 35, 159–168. doi: 10.1007/s12144-015-9378-y

[B15] DoanN. PatteK. A. FerroM. A. LeatherdaleS. T. (2020). Reluctancy towards help-seeking for mental health concerns at secondary school among students in the COMPASS study. Int. J. Environ. Res. Public Health 17:7128. doi: 10.3390/ijerph1719712833003499 PMC7579088

[B16] DockseyA. E. GrayN. S. DaviesH. B. SimkissN. SnowdenR. J. (2022). The stigma and self-stigma scales for attitudes to mental health problems: psychometric properties and its relationship to mental health problems and absenteeism. Health Psychol. Res. 10:35630. doi: 10.52965/001c.3563035928586 PMC9346955

[B17] ElshaikhU. SheikR. SaeedR. K. M. ChiveseT. Alsayed HassanD. (2023). Barriers and facilitators of older adults for professional mental health help-seeking: a systematic review. BMC Geriatr. 23:516. doi: 10.1186/s12877-023-04229-x37626290 PMC10463345

[B18] Essau C. A. (2005). Frequency and patterns of mental health services utilization among adolescents with anxiety and depressive disorders. Depress. Anxiety 22, 130–137. doi: 10.1002/da.20115 16175563

[B19] Faul F. Erdfelder E. Buchner A. Lang A. G. (2009). Statistical power analyses using G^*^Power 3.1: tests for correlation and regression analyses. Behav. Res. Methods 41, 1149–1160. doi: 10.3758/BRM.41.4.1149 19897823

[B20] Foster S. Carvallo M. Lee J. Bernier I. (2021). Honor and seeking mental health services: the roles of stigma and reputation concerns. J. Cross-Cult. Psychol. 52, 178–183. doi: 10.1177/0022022120982070

[B21] Galán-VeraI. Z. LievoreR. MoscardinoU. MammarellaI. C. (2025). Cross-cultural perspectives on social difficulties and anxiety in youth: the role of family cultural values in Mexico and Italy. Front. Psychol. 16:1680963. doi: 10.3389/fpsyg.2025.168096341341732 PMC12671049

[B22] Gambrel P. A. Cianci R. (2003). Maslow's hierarchy of needs: does it apply in a collectivist culture? J. Appl. Manag. Entrep. 8, 143–161.

[B23] Hammer J. H. Spiker D. A. (2018). Dimensionality, reliability, and predictive evidence of validity for three help-seeking intention instruments: ISCI, GHSQ, and MHSIS. J. Couns. Psychol. 65, 394–401. doi: 10.1037/cou0000256 29672088

[B24] Heerde J. A. Hemphill S. A. (2018). Examination of associations between informal help-seeking behavior, social support, and adolescent psychosocial outcomes: a meta-analysis. Dev. Rev. 47, 44–62. doi: 10.1016/j.dr.2017.10.001

[B25] Hofstede G. (2001). Culture's Consequences: Comparing Values, Behaviors, Institutions, and Organizations across Nations, 2nd Edn. Thousand Oaks, CA: Sage.

[B26] Hwang W. C. Myers H. F. Abe-Kim J. Ting J. Y. (2008). A conceptual paradigm for understanding culture's impact on mental health: the cultural influences on mental health (CIMH) model. Clin. Psychol. Rev. 28, 211–227. doi: 10.1016/j.cpr.2007.05.001 17587473

[B27] Isaak C. A. Mota N. Medved M. Katz L. Y. Elias B. Mignone J. . (2020). Conceptualizations of help-seeking for mental health concerns in first nations communities in Canada: a comparison of fit with the Andersen behavioral model. Transcult. Psychiatry 57, 346–362. doi: 10.1177/1363461520906978 32116153

[B28] Jarvis J. A. Corbett A. W. Thorpe J. D. Dufur M. J. (2020). Too much of a good thing: Social capital and academic stress in South Korea. Soc. Sci. 9:187. doi: 10.3390/socsci9110187

[B29] Joshanloo M. (2014). Eastern conceptualizations of happiness: fundamental differences with Western views. J. Happiness Stud. 15, 475–493. doi: 10.1007/s10902-013-9431-1

[B30] Jost J. T. Glaser J. Kruglanski A. W. Sulloway F. J. (2004). Political conservatism as motivated social cognition. Psychol. Bull. 129, 339–375. doi: 10.1037/0033-2909.129.3.339 12784934

[B31] Kim E. Lee H. A. Lee Y. R. Lee I. S. Na K. S. Ahn S. H. . (2025). A new agenda for optimizing roles and infrastructure in a mental health service model for South Korea. Psychiatry Investig. 22, 26–39. doi: 10.30773/pi.2024.0189 PMC11788841 39885789

[B32] Kito M. Yuki M. Thomson R. (2017). Relational mobility and close relationships: a socioecological approach to explain cross-cultural differences. Pers. Relatsh. 24, 114–130. doi: 10.1111/pere.12174

[B33] KocaleventR. D. BergL. BeutelM. E. HinzA. ZengerM. HärterM. . (2018). Social support in the general population: standardization of the Oslo Social Support Scale (OSSS-3). BMC Psychol. 6:31. doi: 10.1186/s40359-018-0249-930016997 PMC6050647

[B34] Krokstad S. Weiss D. A. Krokstad M. A. Rangul V. Kvaløy K. Ingul J. M. . (2022). Divergent decennial trends in mental health according to age reveal poorer mental health for young people: repeated cross-sectional population-based surveys from the HUNT Study, Norway. BMJ Open 12:e057654. doi: 10.1136/bmjopen-2021-057654 PMC9119156 35584877

[B35] KrzyżE. Z. Antunez MartinezO. F. LinH. R. (2023). Uses of Andersen health services utilization framework to determine healthcare utilization for mental health among migrants: a scoping review. Front. Public Health 11:1284784. doi: 10.3389/fpubh.2023.128478438170142 PMC10761300

[B36] Leong F. T. Lau A. S. (2001). Barriers to providing effective mental health services to Asian Americans. Ment. Health Serv. Res. 3, 201–214. doi: 10.1023/A:1013177014788 11859966

[B37] Levesque A. Li H. Z. (2014). The relationship between culture, health conceptions, and health practices: a qualitative–quantitative approach. J. Cross-Cult. Psychol. 45, 628–645. doi: 10.1177/0022022113519855

[B38] LimN. (2016). Cultural differences in emotion: differences in emotional arousal level between the East and the West. Integr. Med. Res. 5, 105–109. doi: 10.1016/j.imr.2016.03.00428462104 PMC5381435

[B39] Luciano M. Sampogna G. Del Vecchio V. Giacco D. Mulè A. De Rosa C. . (2012). The family in Italy: cultural changes and implications for treatment. Int. Rev. Psychiatry 24, 149–156. doi: 10.3109/09540261.2012.656306 22515465

[B40] Markus H. R. Kitayama S. (1991). Culture and the self: implications for cognition, emotion, and motivation. Psychol. Rev. 98, 224–253. doi: 10.1037/0033-295X.98.2.224

[B41] McKenzieS. K. OliffeJ. L. BlackA. CollingsS. (2022). Men's experiences of mental illness stigma across the lifespan: a scoping review. Am. J. Mens Health 16:15579883221074789. doi: 10.1177/1557988322107478935125015 PMC8832600

[B42] MojaverianT. HashimotoT. KimH. S. (2013). Cultural differences in professional help-seeking: a comparison of Japan and the US. Front. Psychol. 3:615. doi: 10.3389/fpsyg.2012.0061523426857 PMC3576055

[B43] Möller-Leimkühler A. M. (2002). Barriers to help-seeking by men: a review of sociocultural and clinical literature with particular reference to depression. J. Affect. Disord. 71, 1–9. doi: 10.1016/S0165-0327(01)00379-2 12167495

[B44] Office of the Surgeon General (US) Center for Mental Health Services (US), and National Institute of Mental Health (US),. (2001). Mental Health: Culture, Race, and Ethnicity: A Supplement to Mental Health: A Report of the Surgeon General. Rockville, MD: Substance Abuse and Mental Health Services Administration (US). 20669516

[B45] Obasi E. M. Leong F. T. (2009). Psychological distress, acculturation, and mental health-seeking attitudes among people of African descent in the United States: a preliminary investigation. J. Couns. Psychol. 56, 227–238. doi: 10.1037/a0014865

[B46] OkaforI. P. OyewaleD. V. OhazurikeC. OgunyemiA. O. (2022). Role of traditional beliefs in the knowledge and perceptions of mental health and illness amongst rural-dwelling women in western Nigeria. Afr. J. Prim. Health Care Fam. Med. 14, e1–e8. doi: 10.4102/phcfm.v14i1.354736226933 PMC9575354

[B47] Omori M. (2007). Japanese college students' attitudes toward professional psychological services: the role of cultural self-construal and self-concealment. Psychol. Rep. 100, 387–399. doi: 10.2466/pr0.100.2.387-399 17564213

[B48] Park M. Chesla C. (2007). Revisiting confucianism as a conceptual framework for Asian family study. J. Fam. Nurs. 13, 293–311. doi: 10.1177/1074840707304400 17641110

[B49] Pescosolido B. A. Boyer C. A. (2010). “Understanding the context and dynamic social processes of mental health treatment,” in A Handbook for the Study of Mental Health, 2nd Edn, eds. T. L. Scheid and T. N. Brown (Cambridge: Cambridge University Press), 420–438. doi: 10.1017/CBO9780511984945.026

[B50] QiuL. XuH. LiY. (2024). Gender differences in attitudes towards psychological help-seeking among Chinese medical students: a comparative analysis. BMC Public Health 24:1314. doi: 10.1186/s12889-024-18826-x38750484 PMC11095043

[B51] Radhamony R. Cross W. M. Townsin L. Banik B. (2024). Culturally and linguistically diverse community access and utilisation of the mental health service: an explanation using Andersen's behavioural model. Issues Ment. Health Nurs. 45, 758–765. doi: 10.1080/01612840.2024.2359602 38954511

[B52] Rickwood D. Deane F. P. Wilson C. J. Ciarrochi J. (2005). Young people's help-seeking for mental health problems. Australas. Psychiatry 4, 218–251. doi: 10.5172/jamh.4.3.218

[B53] RimS. J. HahmB. J. SeongS. J. ParkJ. E. ChangS. M. KimB. S. . (2023). Prevalence of mental disorders and associated factors in Korean adults: National Mental Health Survey of Korea 2021. Psychiatry Investig. 20, 262–272. doi: 10.30773/pi.2022.030736990670 PMC10064208

[B54] Roh S. Burnette C. E. Lee K. H. Lee Y. Easton S. D. Lawler M. J. (2015). Risk and protective factors for depressive symptoms among American Indian older adults: adverse childhood experiences and social support. Aging Ment. Health 19, 371–380. doi: 10.1080/13607863.2014.938603 25070293

[B55] Rosseel Y. (2012). lavaan: an R package for structural equation modeling. J. Stat. Softw. 48, 1–36. doi: 10.18637/jss.v048.i02

[B56] Rüsch N. Angermeyer M. C. Corrigan P. W. (2005). Mental illness stigma: concepts, consequences, and initiatives to reduce stigma. Eur. Psychiatry 20, 529–539. doi: 10.1016/j.eurpsy.2005.04.004 16171984

[B57] Santos H. C. Varnum M. E. W. Grossmann I. (2017). Global increases in individualism. Psychol. Sci. 28, 1228–1239. doi: 10.1177/0956797617700622 28703638

[B58] Schwartz S. H. (2014). Rethinking the concept and measurement of societal culture in light of empirical findings. J. Cross-Cult. Psychol. 45, 5–15. doi: 10.1177/0022022113490830

[B59] Scott G. Ciarrochi J. Deane F. P. (2004). Disadvantages of being an individualist in an individualistic culture: idiocentrism, emotional competence, stress, and mental health. Aust. Psychol. 39, 143–154. doi: 10.1080/00050060410001701861

[B60] SeoH. Y. SongG. Y. KuJ. W. ParkH. Y. MyungW. KimH. J. . (2022). Perceived barriers to psychiatric help-seeking in South Korea by age groups: text mining analyses of social media big data. BMC Psychiatry 22:332. doi: 10.1186/s12888-022-03969-135562709 PMC9102713

[B61] SeyfiF. PoudelK. C. YasuokaJ. OtsukaK. JimbaM. (2013). Intention to seek professional psychological help among college students in Turkey: influence of help-seeking attitudes. BMC Res. Notes 6:519. doi: 10.1186/1756-0500-6-51924313965 PMC4028976

[B62] Shimada S. Tokiwa Y. Iijima Y. (2021). The process by which mothers of one- to two-month-old infants seek help from nursing professionals. Kitakanto Med. J. 71, 93–104. doi: 10.2974/kmj.71.93

[B63] Singelis T. M. Brown W. J. (1995). Culture, self, and collectivist communication: linking culture to individual behavior. Hum. Commun. Res. 21, 354–389. doi: 10.1111/j.1468-2958.1995.tb00351.x 12349710

[B64] Taylor S. E. Sherman D. K. Kim H. S. Jarcho J. Takagi K. Dunagan M. S. (2004). Culture and social support: Who seeks it and why? J. Pers. Soc. Psychol. 87, 354–362. doi: 10.1037/0022-3514.87.3.354 15382985

[B65] Triandis H. C. (2018). Individualism and Collectivism. New York, NY: Routledge. doi: 10.4324/9780429499845

[B66] Vogel D. L. Heimerdinger-Edwards S. R. Hammer J. H. Hubbard A. (2011). 'Boys don't cry': examination of the links between endorsement of masculine norms, self-stigma, and help-seeking attitudes for men from diverse backgrounds. J. Couns. Psychol. 58, 368–382. doi: 10.1037/a0023688 21639615

[B67] Vogel D. L. Wei M. (2005). Adult attachment and help-seeking intent: the mediating roles of psychological distress and perceived social support. J. Couns. Psychol. 52, 347–357. doi: 10.1037/0022-0167.52.3.347

[B68] White K. R. Kinney D. Danek R. H. Smith B. Harben C. (2020). The resistance to change-beliefs scale: validation of a new measure of conservative ideology. Pers. Soc. Psychol. Bull. 46, 20–35. doi: 10.1177/0146167219841624 31064288

[B69] YangL. H. KleinmanA. (2008). 'Face' and the embodiment of stigma in China: the cases of schizophrenia and AIDS. Soc. Sci. Med. 67, 398–408. doi: 10.1016/j.socscimed.2008.03.01118420325 PMC2911783

[B70] Yeh C. J. (2002). Taiwanese students' gender, age, interdependent and independent self-construal, and collective self-esteem as predictors of professional psychological help-seeking attitudes. Cult. Divers. Ethn. Minor. Psychol. 8, 19–29. doi: 10.1037/1099-9809.8.1.19 12092425

